# Remote control of magnetostriction-based nanocontacts at room temperature

**DOI:** 10.1038/srep13621

**Published:** 2015-09-01

**Authors:** S. Narayana Jammalamadaka, Sebastian Kuntz, Oliver Berg, Wolfram Kittler, U. Mohanan Kannan, J. Arout Chelvane, Christoph Sürgers

**Affiliations:** 1 Magnetic Materials and Device Physics Laboratory, Department of Physics, Indian Institute of Technology Hyderabad, Hyderabad 502 205, India; 2 Physikalisches Institut, Karlsruhe Institute of Technology, Wolfgang Gaede Str. 1, Karlsruhe, 76131, Germany; 3 Defence Metallurgical Research Laboratory, Hyderabad 500058, India

## Abstract

The remote control of the electrical conductance through nanosized junctions at room temperature will play an important role in future nano-electromechanical systems and electronic devices. This can be achieved by exploiting the magnetostriction effects of ferromagnetic materials. Here we report on the electrical conductance of magnetic nanocontacts obtained from wires of the giant magnetostrictive compound Tb_0.3_Dy_0.7_Fe_1.95_ as an active element in a mechanically controlled break-junction device. The nanocontacts are reproducibly switched at room temperature between “open” (zero conductance) and “closed” (nonzero conductance) states by variation of a magnetic field applied perpendicularly to the long wire axis. Conductance measurements in a magnetic field oriented parallel to the long wire axis exhibit a different behaviour where the conductance switches between both states only in a limited field range close to the coercive field. Investigating the conductance in the regime of electron tunneling by mechanical or magnetostrictive control of the electrode separation enables an estimation of the magnetostriction. The present results pave the way to utilize the material in devices based on nano-electromechanical systems operating at room temperature.

The ability to manipulate and remotely control the electronic transport through single-atom or few-atom contacts is currently of great interest due to their potential applications in electronic devices^1^ and nanoelectromechanical systems (NEMS)^2^. In particular, mechanically-controlled break junctions^3^,^4^, scanning tunneling microscopy^5^, and electrochemical methods^6^ have extensively been used to investigate the electronic transport through monatomic contacts. A mechanically-controlled break junction (MCBJ) is an electronic device where the distance between two metal electrodes can be tuned by bending the substrate to establish a contact comprising only a few or a single atom^3^,^4^. Several groups have reported interesting results on magnetic nano-contacts such as low-temperature magnetoresistance in ferromagnetic break junctions^7^,^8^, conductance quantization in nickel (Ni) nanowires^9^, tuning of magnetoresistance in nanocontacts by magnetostriction^10^, and magnetoresistance of a single nickel atom^11^. Controlling the electrical conductance *G* in the range of a few *G*_0_, where 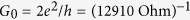 is the conductance quantum, by an applied magnetic field *H* rather by mechanical control has been demonstrated recently by exploiting the large magnetostriction of the rare-earth element dysprosium at low temperatures^12^.

Magnetostriction is a property of a ferromagnetic material that changes the volume of the material due to magnetic order. The magnetostrictive strain – the relative length change *λ* = Δ*l*/*l* in the direction of the magnetization – is associated with the magnetization process and depends on the applied magnetic field^13^,^14^,^15^,^16^,^17^,^18^,^19^. Typical values for the ferromagnets Fe, Co, and Ni are in the range of a few 10^−6^ at room temperature. Extensive work has been carried out to find a compound which exhibits a giant magnetostriction with low anisotropy at room temperature^13^,^14^,^15^,^16^,^17^,^18^,^19^. Among these compounds, the rare-earth transition-metal compound (Tb_0.27_Dy_0.73_)Fe_1.95_ with cubic structure is commercially available as rods under the name Terfenol-D^18^. The material properties can be further tuned to obtain the anisotropy-compensated compound Tb_0.3_Dy_0.7_Fe_1.95_ with low first-order magnetocrystalline anisotropy constant *K*_1_ = −6 × 10^4^Jm^−3^ and huge magnetostriction *λ* = 1.6 × 10^−3^ at room temperature^20^. This material can be used in the form of wires to realize a magnetic-field operated “nanoswitch” at room temperature. However, the machining of this material is extremely difficult as its Young’s modulus is extremely low^16^,^17^.

Conductance measurements have been reported for single-element based atomic junctions but not for junctions comprising multi-element compounds^21^,^22^, in particular not for devices exploiting giant-magnetostriction phenomena at room temperature. Since Tb_0.3_Dy_0.7_Fe_1.95_ has a giant magnetostrictive strain at room temperature, the gap between single atoms or few atoms could be controlled in a remote way without mechanical access, which would be beneficial compared to conventional MCBJ devices. In the present work, we have realized the aforementioned ideas on Tb_0.3_Dy_0.7_Fe_1.95_ based nano-contact devices. The contacts can be switched at room temperature by a magnetic field and the observed behaviour is in qualitative agreement with the magnetization process. The conductance switching strongly depends on the field orientation with respect to the contact due to the magnetic anisotropy. Comparison of the electron tunneling data when controlling the gap between two contacts either mechanically or by magnetic field allows an estimation of the magnetostriction of Tb_0.3_Dy_0.7_Fe_1.95_.

## Results

### Characterization of Tb_0.3_Dy_0.7_Fe_1.95_

**Figure 1:**
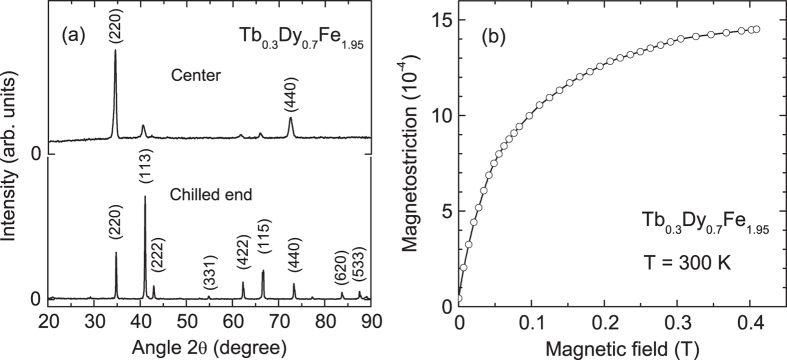
Texture and magnetostriction of Tb_0.3_Dy_0.7_Fe_1.95_. **(a)** X-ray diffraction pattern (Cu K_*α*_ radiation) recorded with scattering vector oriented along the long axis of the rod. The data obtained at the chilled end (bottom) show a polycrystalline structure while the center part 5 cm away from the chilled end, which was slowly removed from the hot zone (top), has a strong fiber texture along the 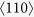 direction. **(b)** Magnetostriction at room temperature measured with a strain gauge vs. magnetic field oriented along the long axis of the rod.

A Tb_0.3_Dy_0.7_Fe_1.95_ rod was obtained by employing a modified Bridgman method for crystal growth, see Methods section. Figure 1(a) shows the x-ray diffraction (XRD) pattern of two pieces of material. The data obtained from a piece cut from the chilled end of the rod (bottom) indicate a polycristalline structure, while the piece cut from the part slowly retracted from the hot zone 5 cm from the chilled end (top) has a preferred orientation with the crystallographic 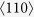 axes along the rod axis. The 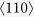 direction makes an angle of 35 degrees with the 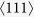 direction which is the magnetic easy-axis^16^,^18^,^19^,^20^. Pieces cut from this directionally solidified part of the rod were used for the conductance and magnetostriction measurements. The magnetostriction vs. magnetic field *H* is plotted in Fig. 1(b) which confirms a giant value of *λ* ≈ 1.5 × 10^−3^ in 0.4 Tesla at room temperature.

### The MCBJ device

**Figure 2:**
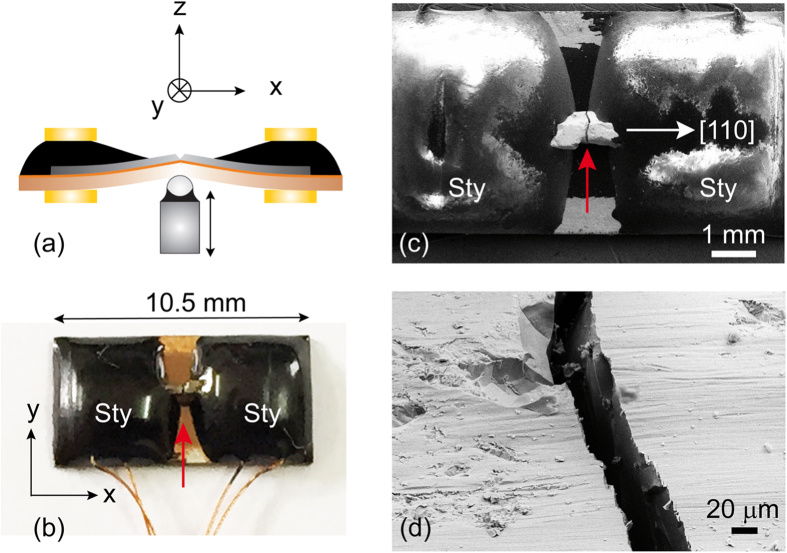
Mechanically-controlled break junction of Tb_0.3_Dy_0.7_Fe_1.95_. **(a)** Schematic (side view) of the Tb_0.3_Dy_0.7_Fe_1.95_ wire (grey) glued with Stycast epoxy (black) to the Cu bronze substrate (brown) fixed between metallic supports (yellow). The substrate can be bended by pushing the piezo-driven rod from below. **(b)** Photograph (top view) of a 8-mm long Tb_0.3_Dy_0.7_Fe_1.95_ sample with Cu wires attached. The sample and the electrical contacts are almost completely covered by Stycast epoxy (Sty) apart from the inner 2 mm where the material breaks (red arrow) and forms a junction. **(c)** SEM image of the 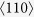-oriented wire (top view). Red arrow indicates the junction, Sty indicates the Stycast epoxy drops. **(d)** SEM image of the junction seen under an oblique angle. The SEM images show a clear rupture of the order of 20 *μ*m at the junction between the electrodes due to mechanical breaking.

Figure 2(a) shows a schematic of the MCBJ device. The material under study (grey) is fixed to a flexible substrate which can be bended mechanically by pushing a piston against the back of the substrate. Fine tuning of the bending is achieved by using a voltage controlled piezo stack^12^. In the present case, Tb_0.3_Dy_0.7_Fe_1.95_ thin wires of ≈ 1 × 1 mm^2^ cross section and 8 mm length were obtained by carefully shaping appropriate pieces with emery paper and subsequent cleaning by ultrasonic vibration. Copper wires were connected to the sample by conductive silver epoxy Epo-Tek H20E to perform four-point conductance measurements. The sample with attached Cu wires was almost completely covered with Stycast 2850FT epoxy and glued to a flexible (5.5 × 10.5 mm^2^), 0.3 mm thick copper-bronze substrate coated with a 2 *μ*m thick durimide film for electrical insulation, see Fig. 2(b,c). A notch was created in the inner region (≈ 2 mm) not covered by Stycast epoxy in order to predefine the position where the wire breaks during bending the substrate. The magnetic field was oriented along the z direction in measurements at room temperature or along the x or y direction in measurements at *T* = 4.2 K.

### Switching the contact at room temperature

**Figure 3:**
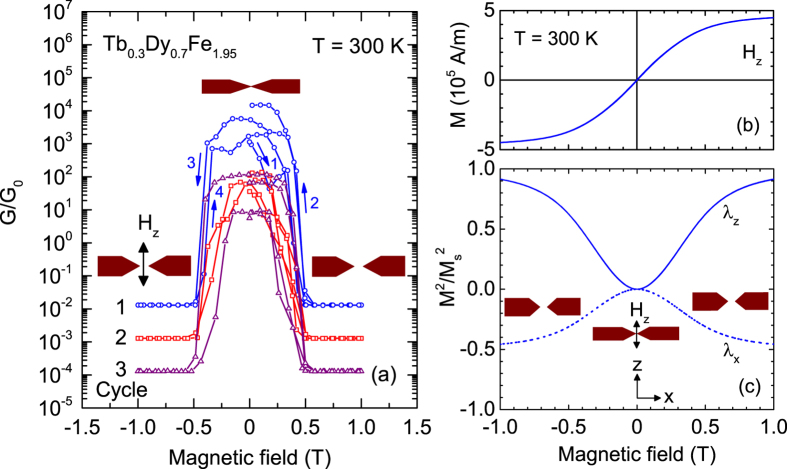
Magnetostriction-controlled conductance switching of Tb_0.3_Dy_0.7_Fe_1.95_ at room temperature. **(a)** Semi-logarithmic plot of the conductance *G* in a magnetic field *H*_*z*_ applied along the z axis. Cycles 2 and 3 have been successively shifted downward by one decade with respect to cycle 1 for clarity. Cartoons visualize the contact configuration in magnetic field due to magnetostriction. **(b)** Magnetization *M* vs. *H*_*z*_. **(c)** 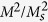 calculated from *M*(*H*_*z*_) (solid line) shows the qualitative behaviour of the magnetostrictive strain *λ*_*z*_ in *H*_*z*_. The corresponding strain along the wire axis (dashed line) is approximated by *λ*_*x*_ = −*λ*_*z*_/2.

Figure 3(a) shows the electrical conductance *G* vs. magnetic field *H* for a grain oriented Tb_0.3_Dy_0.7_Fe_1.95_ break junction at room temperature with the magnetic field applied in the z direction. Initially, the junction was adjusted to be in weak contact at *G* ≈ 100–1000 *G*_0_ by mechanically bending the substrate. This conductance corresponds to a junction with a contact diameter of a few nm^3^. After a few cycles of initial switching without hysteresis the contact reproducibly switches from a “closed” (*G* *>* 0) to an “open” (*G* *=* 0) state when the magnetic field increases from zero to above 0.5 T. The finite conductance of ≈ 10^−2^*G*/*G*_0_ measured in the open state is due to the 1 MΩ resistor connected in parallel to the device, see Methods section. The arrows in the graph indicate the evolution of the conductance during the field sweep. The device configuration at each state is visualized by cartoons (brown colour). This switching behaviour of the conductance was measured several times and was established on several samples. The field dependence of the magnetization *M* in a magnetic field *H*_*z*_ oriented perpendicularly to the long wire axis shows a hard-axis behaviour, see Fig. 3(b).

From 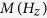 the qualitative behaviour of the magnetostrictive strains *λ*_*z*_ and *λ*_*x*_ in the z and x direction, respectively, are obtained. Usually, the uniaxial magnetostrictive strain along a hard axis is roughly proportional to the square of *M*, i.e., *λ*/*λ*_*s*_ = *M*^2^/*M_s_*^2^, where *M*_*s*_ and *λ*_*s*_ > 0 are the saturation values measured in a high magnetic field of 2 T^17^. The field dependence of *λ*_*z*_ is shown in Fig. 3(c) (solid line). However, the conductance of the device depends only on the electrode separation along the x direction which is affected by magnetostriction. For polycrystals, the magnetostriction in the x direction perpendicular to the magnetization is approximated by *λ*_*x*_/*λ*_*xs*_ = −*λ*_*z*_/2*λ*_*zs*_ shown in Fig. 3(c) (dashed line)^17^. Hence, starting from a closed contact in zero field the electrodes should increase in diameter along the z direction and shrink their length along the x direction with increasing field due to the positive magnetostriction of the material. This leads to an opening of the contact in high magnetic fields as observed in Fig. 3(a).

### Switching the contact at different temperatures

**Figure 4:**
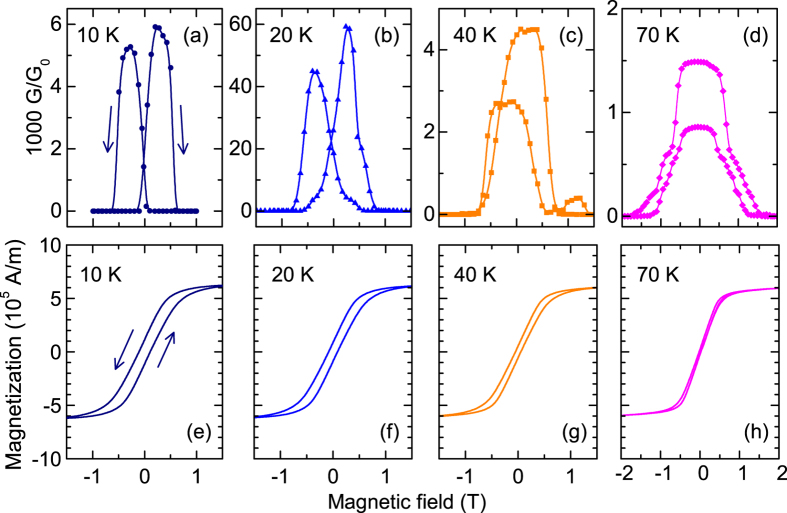
Temperature dependence of magnetostriction-controlled conductance switching of Tb_0.3_Dy_0.7_Fe_1.95_. **(a–d)** Conductance vs. magnetic field applied perpendicularly to the long wire axis. **(e–h)** Magnetization curves in perpendicular magnetic field at various temperatures. A hysteresis loop opens below 70 K in accordance with the hysteresis observed in the conductance switching.

The effect of temperature on the switching of the conductance *G*(*H*) was investigated in the temperature range 10–300 K. Figure 4(a–d) shows exemplary *G(H)* curves of the Tb_0.3_Dy_0.7_Fe_1.95_ break junction at various temperatures. We observe a switching between high conductance and low conductance at each temperature. In these measurements, the contact was initially adjusted in an “open” state in high magnetic field and then the field was reduced. Hence, the maximum of *G* depends on the arbitrary electrode separation before performing the field sweep. The difference in the maxima between up and down sweeps is attributed to changes of the nanocontact configuration when the contact opens and closes several times during performing a complete field loop.

At temperatures below 70 K we observe a hysteresis of the switching in *G(H)* which is also seen in the magnetization curves *M*(*H*) shown in Fig. 4(e–h). However, the maxima in *G(H)* [Fig. 4(a,b)] occur at higher magnetic fields than the coercive fields in *M*(*H*) [Fig. 4(e,f)]. This is presumably due to deviations of the local micromagnetic structure close to the nanocontact from the volume-integrated magnetization *M*(*H*) measured by the magnetometer. Nevertheless, the qualitative behaviour between *G(H)* and *M*(*H*) is the same regarding the occurrence of a hysteresis below 70 K. The reason for the hysteresis might be a pinning of magnetic domain walls. In Ni-substituted Dy_0.73_Tb_0.27_Fe_2_, a maximum in the temperature dependence of the magnetization in 1 T for *T* *<* 50 K was attributed to the strong pinning of domain walls at *T* = 4.2 K and the decrease of the pinning barrier with increasing temperature^23^. This suggests that a similar effect of domain wall pinning gives rise to the hysteresis in the magnetization and in the conductance switching of Tb_0.3_Dy_0.7_Fe_1.95_ nanocontacts at temperatures below 70 K which disappears at higher temperatures.

**Figure 5:**
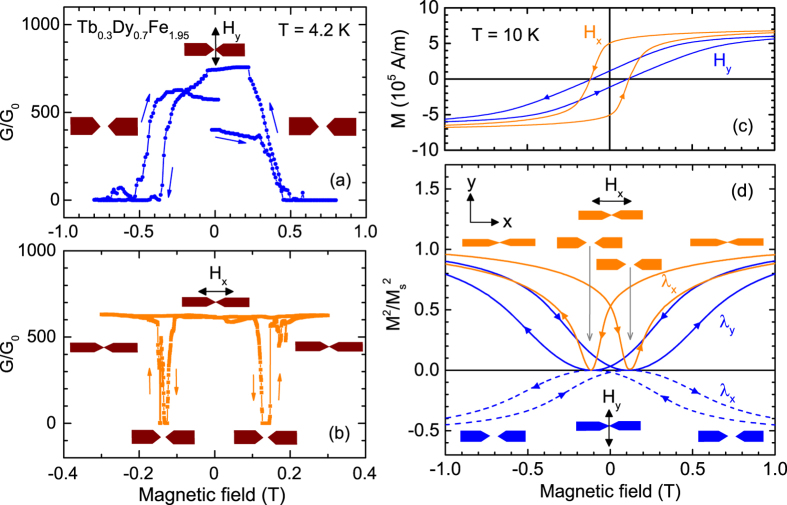
Conductance switching of Tb_0.3_Dy_0.7_Fe_1.95_ at low temperatures in different field orientations. **(a)** Conductance *G*/*G*_0_ in perpendicular field *H*_*y*_ at *T* = 4.2 K. **(b)** Conductance *G*/*G*_0_ in parallel field *H*_*x*_ at *T* = 4.2 K. **(c)** Magnetization loops obtained at *T* = 10 K. **(d)** Qualitative behaviour of the magnetostrictive strains *λ*_*x*_ and *λ*_*y*_ calculated from 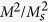 in *H*_*x*_ and *H*_*y*_ (solid lines), see text for details. In perpendicular field *H*_*y*_, the strain along the long wire axis is *λ*_*x*_ = −*λ*_*y*_/2 (dashed line). Cartoons visualize the contact configuration in magnetic field due to magnetostriction.

We now focus on the conductance switching performed at constant temperature *T* = 4.2 K for different orientations of the magnetic field to explore the effect of magnetic anisotropy on the switching behaviour. The magnetic field could be rotated in the x-y plane of the sample either parallel (x) or perpendicular (y) to the long wire axis. In nanocontact devices, a hysteresis of *M(H)* can cause negative magnetostrain, which would eventually alter the magnetostriction behaviour *λ*(*H*)^12^. To avoid such effects, we demagnetized the contact before we started the field sweep. Figure 5(a) shows a measurement performed at *T* = 4.2 K with the magnetic field *H*_*y*_ oriented perpendicularly to the long wire axis (y direction). In this configuration, the device was in the “closed” state before application of the magnetic field. With increasing field *H*_*y*_ the conductance drops at a magnetic field of 0.4–0.5 T and the contact opens due to the extension of the wire diameter along the field (y direction) and the corresponding shrinkage along the wire axis (x direction) due to magnetostriction. In addition, we observe a hysteresis in the switching at negative field due to a hysteresis in the magnetization curve shown in Fig. 5(c) with a coercivity *μ*_0_*H*_*c*_ = 0.12 T. For this sample a corresponding hysteresis at positive fields is missing. One reason for this asymmetric behaviour might be the different magnetic domain configurations when the magnetic field is rotated by 180°. We calculate the field dependence of the magnetostrictive strain from the magnetization curve *M*(*H*_*y*_) of Fig. 5(c) in both directions as mentioned above, see Fig. 5(d) (blue curves). The behaviour of the magnetostrictive strain *λ*_*x*_(*H*_*y*_)/*λ*_*xs*_ along the long wire axis x (dashed line) resembles the behaviour of *G*(*H*_*y*_). The smooth variation of the magnetostrain with increasing field towards negative values along the x direction gives rise to an increasing separation between the two electrodes until the contact opens. The closing of the contact around zero field is in agreement with the *M*^2^(*H*_*y*_) behaviour, see Fig. 5(d) (dashed curve).

In a magnetic field *H*_*x*_ applied parallel to the long wire axis a different switching behaviour is observed, see Fig. 5(b). Starting from a “closed” state and increasing the field, the conductance is almost constant up to ≈0.1 T where *G* sharply drops to zero representing the opening of the contact. By increasing the field further to ≈0.15 T the conductance suddenly recovers and reaches its zero-field value at high fields indicating a closed contact. Reducing the field subsequently and sweeping to negative field values does not change the conductance until between −0.1 and −0.15 T the same behaviour is observed like for positive fields. The switching behaviour with two sharp dips of *G*(*H*) at ≈ ±0.15 T is in qualitative agreement with the behaviour of the magnetization and corresponding magnetostrictive strain. Figure 5(c) shows the magnetization of the sample vs. field *H*_*x*_ representing a more rectangular hysteresis loop with a coercive field *μ*_0_*H*_*c*_ = 0.12 T, similar to *H*_*c*_ obtained in perpendicular field *H*_*y*_. The different shape of *M*(*H*) in the two field orientations demonstrates the magnetic anisotropy of the 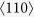-oriented sample. The almost rectangular loop gives rise to a strong field dependence of the magnetostrain derived from 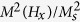 plotted in Fig. 5(d) (yellow curve). In particular, the magnetostrain is almost zero close to the coercive field due to the reorientation of magnetic domains and *M* = 0 at *H*_*c*_. However, above and below *H*_*c*_, the magnetostrain is large due to the strong variation of *M*(*H*_*x*_) which gives rise to two dips in *λ*_*x*_ close to *H*_*c*_. This field dependence immediately explains the observed switching of the nanocontact in parallel field *H*_*x*_ [Fig. 5(b)]. Starting at a closed state at zero field after magnetizing the sample, the magnetostrain along the x direction strongly drops close to *H*_*c*_ leading to an opening of the contact. After the reorientation of domains for fields *H*_*x*_ > *H*_*c*_ the magnetostrain rises and the contact closes again. This is supported by the fact that the opening of the contact and the drop of conductance start to occur at *H*_*c*_, cf. Fig. 5(b). Hence, the magnetic anisotropy of the material strongly affects the magnetostriction-controlled switching behaviour of the break junction in different orientations of the magnetic field.

### Estimation of the magnetostriction from the tunneling conductance

Once the contact has been adjusted to exhibit a conductance well below *G*_0_, the electronic transport is dominated by tunneling. The tunneling current depends on the gap between the two electrodes which can be controlled mechanically or by magnetostriction. The comparison of the tunneling behaviour observed in both cases allows an estimation of the magnetostriction as we demonstrate in the following. However, in the present case the tunneling behaviour could only be observed at low temperatures.

**Figure 6:**
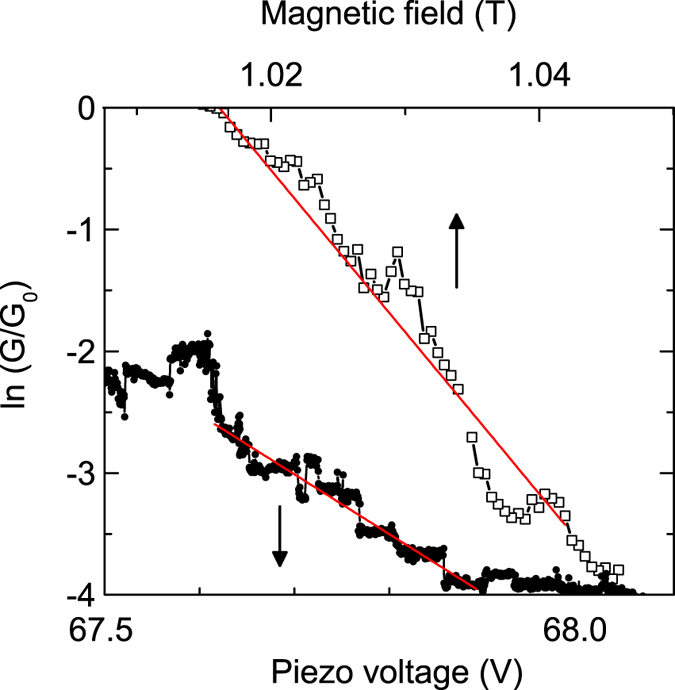
Conductance in the regime of electron tunneling. Semilogarithmic plot of *G*/*G*_0_ vs. piezo voltage or magnetic field applied perpendicularly to the long wire axis at *T* = 10 K. Red lines indicate a linear dependence ln(*G*/*G*_0_) ∝ *V* or ln(*G*/*G*_0_) ∝ *H*, respectively.

Figure 6 shows ln (*G*/*G*_0_) vs. piezo voltage, i.e., electrode separation along the x direction, for 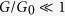 at *T* = 10 K indicating an exponential decay of the conductance with electrode separation Δ*x* characteristic for electron tunneling


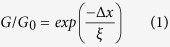


where 
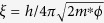
, *h* is the Planck constant, m^*^ is effective electron mass, and *ϕ* is the average electronic work function of Tb_0.3_Dy_0.7_Fe_1.95_. An average work function *ϕ* = 5.9 eV and *ξ* = 0.4 Å was used for Tb_0.3_Dy_0.7_Fe_1.95_ by considering the individual work functions of the constituents and by taking into account the change of the work function in helium atmosphere^24^. From the linear slope 1/*ξ*_*V*_ of the semilogarithmic plot of *G(V)* in Fig. 6 and considering Δ*x*/*ξ* = Δ*V*/*ξ*_*V*_ we estimate a variation of the gap by the piezo voltage to 2.0 Å/V.

A similar calculation can be performed when the gap is controlled by magnetostriction. Figure 6 shows ln(*G*/*G*_0_) vs. perpendicular magnetic field at 10 K. Again, the *G(H)* data measured during closing show an exponential behaviour characteristic for electron tunnneling with a slope 1/*ξ*_*H*_ in a ln(*G*/*G*_0_) vs. *H* plot^12^,^25^. The variation of the electrode distance, i.e., the gap Δ*x*, by a field variation around *μ*_0_*H* = 1 T is calculated from Δ*x*/*ξ* = *μ*_0_Δ*H*/*ξ*_*H*_ similarly to the mechanical control above. We obtain Δ*x*/*μ*_0_Δ*H* = 5.3 nm/T for the increase of the gap with increasing field, corresponding to a relative shrinkage of the wire length Δ*L*/*L* = −2.7 × 10^−6^ along the x direction (*L* ≈ 2 mm). The observed field-induced gap variation in x direction is caused by the magnetostrain due to the corresponding increase of the wire diameter by −2Δ*L*/*L* = 5.4 × 10^−6^ along the magnetic field in perpendicular direction. From this we estimate the magnetostriction of Tb_0.3_Dy_0.7_Fe_1.95_ at 10 K by


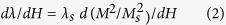


Here, 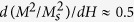/T is the slope of 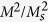 representing *λ* in perpendicular field around *μ*_0_*H* = 1 T [Fig. 5(d) blue curve]. Hence, we obtain a low saturation magnetostriction *λ*_*s*_ ≈ 1 × 10^−5^ for Tb_0.3_Dy_0.7_Fe_1.95_ at 10 K, two orders of magnitude smaller than at room temperature^26^,^27^.

## Discussion

The results clearly demonstrate the remote control of the nanocontact conductance by a magnetic field. We mention that the measurements for Dy nanocontacts reported earlier^12^ were constrained to low temperatures, where Dy is in a ferromagnetic state and has a large magnetostriction. In the present case of Tb_0.3_Dy_0.7_Fe_1.95_ the conductance of the wire switches at room temperature due the giant magnetostrictive property of this material. The magnetocrystalline and shape anisotropy of the wire plays an important role for the switching behaviour. Application of a magnetic field along the magnetic hard axis, i.e., perpendicular to the long wire axis, is beneficial because in this case the wire is strained continuously without hysteresis. In contrast, if the magnetic field is applied along the magnetic easy axis, i.e., along the long wire axis, an abrupt switching is obtained in a limited field range close to the coercive field where the magnetization spontaneously rotates by 180°. The hysteresis observed in the *G(H)* behaviour at low temperature is attributed to the temperature dependence of the magnetization curves and the pinning of the domain walls at low temperatures. Hence, the switching behaviour in parallel and perpendicular field is entirely different due to the different magnetic anisotropy in the two orientations. From the piezo-voltage as well as magnetic-field control of the conductance, we are able to estimate the field-induced length changes of the contact and the magnetostriction of Tb_0.3_Dy_0.7_Fe_1.95_ at low temperatures. In conclusion, we have realized a magnetic-field induced switching of the conductance *G* of break junctions made from the giant magnetostrictive compound Tb_0.3_Dy_0.7_Fe_1.95_ at room temperature. The results are important when developing future devices based on NEMS.

## Methods

A Tb_0.3_Dy_0.7_Fe_1.95_ compound was prepared in a vacuum induction-furnace by casting the liquid alloy into quartz tubes placed over a water-cooled copper plate. The cast rod was then directionally solidified employing a modified Bridgman technique under high vacuum (10^−6^ mbar). During the directional solidification process, the rod was fixed on a retractable water-cooled copper chilling plate which was then slowly retracted from the hot zone (1350° C) at a constant pulling rate of 70 cm/h. Accordingly, cylindrical directionally solidified rods of 10 cm length and 20 mm diameter were prepared. Since the portion which is in touch with the copper plate does not melt we have taken a sample from the center of the rod at a distance 5 cm from the chilled end for device fabrication. Material characterization was done by back-scattered electron microscopy of the directionally solidified compound along the longitudinal direction and by energy dispersive spectroscopy confirming the presence of single phase with uniform composition along the entire sample.

The room-temperature magnetostriction of Tb_0.3_Dy_0.7_Fe_1.95_ was determined on a separate sample cut from the center of the same rod. A commercially available temperature-compensated 120 Ohm Karma-foil strain gauge with low magnetoresistance (Micro-Measurements Group Inc., USA) was directly attached to the sample with Cynoacrylate cement (M-bond 200 or Anabond 202). The leads were soldered to fine copper wires (SWG 38) for resistance measurements with a Wheatstone bridge. The sample with the strain gauge was carefully placed in between the pole pieces of an electromagnet with the magnetic field applied parallel to the rod axis. The relative length change due to magnetostriction Δ*l*/*l* was calculated from Δ*l*/*l* = 4Δ*E*/*VK*, where Δ*E* is the unbalanced bridge voltage, *V* is the excitation voltage and *K* a the gauge calibration factor.

For the conductance measurements, a 1 MΩ resistor was connected in parallel to the MCBJ as a shunt to avoid large voltage spikes across the contact during opening or closing. The assembly was mounted in a MCBJ device, which was inserted into a physical property measurement system (PPMS, Quantum Design) providing a magnetic field in the z direction perpendicular to the substrate surface. Before breaking the wire, the sample chamber was purged many times with helium gas. In addition, a cryopump was connected to the sample chamber to achieve high-to-ultrahigh vacuum conditions. Mechanical instabilities were minimized by tightly fixing both ends of the wire to the nonmagnetic substrate with high-quality stycast. In addition, before collecting data the temperature of the MCBJ was stabilized to about 1 mK a long period of time.

For performing measurements in different field orientations, the MCBJ was inserted into the liquid ^4^He bath (*T* = 4.2 K) of another cryostat equipped with a superconducting Helmholtz coil providing a magnetic field in the x-y plane of the substrate. The field orientation parallel (x direction) or perpendicular (y direction) to the long wire axis could be changed by rotating the MCBJ device around the substrate normal. The wire was mechanically broken by using a pushing rod which was driven by a stepper motor. Fine tuning of the electrode distance was achieved by a voltage-driven pizo stack between the pushing rod and the substrate. The conductance was monitored by measuring the voltage at a constant current of 1 *μ*A.

The magnetization was measured in a vibrating-sample magnetometer (VSM, Oxford Instruments) at different temperatures between 10 K and 300 K for field orientations parallel or perpendicular to the long wire axis. The sample was vibrating at a frequency of 55 Hz with an amplitude of 0.2 mm. The magnetization was calculated from the measured magnetic moment by using a mass density of 9.25 gcm^−3^.

Scanning electron-microscopy (SEM) images were recorded with various magnifications at a beam energy of 20 keV in a Carl Zeiss Supra 40 electron microscope.

## Additional Information

**How to cite this article**: Jammalamadaka, S. N. *et al.* Remote control of magnetostriction-based nanocontacts at room temperature. *Sci. Rep.* **5**, 13621; doi: 10.1038/srep13621 (2015).
